# Using Mendelian randomization provides genetic insights into potential targets for sepsis treatment

**DOI:** 10.1038/s41598-024-58457-1

**Published:** 2024-04-11

**Authors:** Rui Xia, Meng Sun, Jing Yin, Xu Zhang, Jianhua Li

**Affiliations:** 1 Department of Critical Care Medicine, Chongqing University Jiangjin Hospital, Chongqing, 402260 China; 2 Department of Anesthesiology, Union Hospital, Tongji Medical College, Huazhong University of Science and Technology, Wuhan, 430022 China; 3 Affiliated Hospital of Medical School, Nanjing Jinling Hospital, Nanjing University, Nanjing, 210016 China; 4 Center for Reproductive Medicine, Women and Children’s Hospital of Chongqing Medical University, Chongqing, 400013 China; 5 Center for Reproductive Medicine, Chongqing Health Center for Women and Children, Chongqing, 400013 China; 6 Chongqing Reproductive Genetics Institute, Chongqing, 400013 China

**Keywords:** Mendelian randomization, Sepsis, Drug target prediction, Computational biology and bioinformatics, Drug discovery, Genetics, Medical research

## Abstract

Sepsis is recognized as a major contributor to the global disease burden, but there is a lack of specific and effective therapeutic agents. Utilizing Mendelian randomization (MR) methods alongside evidence of causal genetics presents a chance to discover novel targets for therapeutic intervention. MR approach was employed to investigate potential drug targets for sepsis. Pooled statistics from IEU-B-4980 comprising 11,643 cases and 474,841 controls were initially utilized, and the findings were subsequently replicated in the IEU-B-69 (10,154 cases and 454,764 controls). Causal associations were then validated through colocalization. Furthermore, a range of sensitivity analyses, including MR-Egger intercept tests and Cochran's Q tests, were conducted to evaluate the outcomes of the MR analyses. Three drug targets (PSMA4, IFNAR2, and LY9) exhibited noteworthy MR outcomes in two separate datasets. Notably, PSMA4 demonstrated not only an elevated susceptibility to sepsis (OR 1.32, 95% CI 1.20–1.45, p = 1.66E−08) but also exhibited a robust colocalization with sepsis (PPH4 = 0.74). According to the present MR analysis, PSMA4 emerges as a highly encouraging pharmaceutical target for addressing sepsis. Suppression of PSMA4 could potentially decrease the likelihood of sepsis.

## Introduction

Sepsis arises from a dysregulation of the host's response to infection and immunity, ultimately culminating in life-threatening organ dysfunction^1^. With approximately 11 million related deaths per year, accounting for 20% of all global deaths, its treatment imposes an even greater financial burden, with expenditures of $27 billion attributed to sepsis in the United States in 2019 alone^2,3^. This places a substantial strain on the healthcare system. To date, the management of sepsis has primarily relied on antimicrobial therapy, source control, and organ support^4^. Specific and effective therapeutic agents for clinical use remain elusive. However, as extensively drug-resistant pathogens continue to emerge, the pool of antimicrobial drugs available to treat infected patients is dwindling^5^. The search for effective therapeutic agents or prophylactic targets for sepsis is urgent.

Genetics stands as a potent instrument in propelling drug development forward, and medications underpinned by genetic research are more likely to thrive in clinical trials^6–8^. Currently, proteins encoded by “druggable” genes have become drug targets and hold the potential to be targets for small molecules or monoclonal antibodies as well^9,10^. Despite the effectiveness of genome-wide association studies (GWAS) in identifying single nucleotide polymorphisms (SNPs) linked to the risk and advancement of sepsis, it is not reliable for GWAS alone to precisely determine the causal genes and provide direct insights for drug development^11–13^.

Mendelian randomization (MR) stands as a genetic technique that operates by employing a form of random allocation of genetic alleles that influence exposure^14,15^. Much like randomized controlled trials, it can be harnessed to forecast the effectiveness of a drug. Cis-expression quantitative trait loci (cis-eQTL), situated in the genomic vicinity of a drug target gene, are commonly regarded as surrogates that act as regulators influencing gene expression during drug target MR analysis^16^. This analytical approach has been applied in the exploration of drug targets for various diseases^16,17^. However, up to the present, no investigations employing this technique have unearthed potential drug targets and genomic evidence for sepsis.

In the scope of this study, by combining two independent sepsis GWAS datasets and blood eQTL for MR analysis, we identified potential drug targets for the treatment of sepsis. We explored the connection between genetically influenced targetable genes and the susceptibility to sepsis, ultimately putting forth drug targets for sepsis that are substantiated by genetic evidence.

## Methods

### Data sources

A comprehensive set of 4302 druggable genes, categorized under HGNC nomenclature, was identified on the autosomal chromosomes^9^. Given that cis-eQTL were deemed more proximate to the gene of concern within the domain of drug discovery, we systematically acquired cis-eQTL that were entirely statistically significant (with a false discovery rate < 0.05) and located within a ± 1 Mb radius of each probe. The cis-eQTLs were obtained from the vast collection of resources provided by the eQTLGen Consortium and the eQTL meta-analysis, which included the peripheral blood profiles of 31,684 individuals^18^. To create genetic tools that could act as substitutes for the 4302 targetable objectives, we specifically chose cis-eQTL situated within ± 100 kb of the genomic position of each gene, leading to the presence of eQTL for a total of 2405 targetable genes.

Summary-level data for the Sepsis GWAS were sourced from the IEU-B-4980, a substantial biomedical repository and research asset. Within this dataset, there were 11,643 documented cases of sepsis and 474,841 controls^18^. Of the total participants, 54% were female, and 46% were male, with a median age of 58 years for the entire cohort and 60 years for sepsis cases. The sepsis diagnosis adhered to the criteria outlined in the International Classification of Diseases (ICD)-9 and ICD-10 codes as delineated in the previously published Global Burden of Disease (GBD) study^19^. Detailed information concerning the aforementioned GWAS study has been previously documented. Additionally, we integrated summary metrics obtained from the IEU-B-69 to externally validate our findings, which consisted of 10,154 sepsis patients and 454,764 controls (Table S1).

### Mendelian randomization and colocalization

In our MR analyses, we employed the TwoSampleMR R package. To ensure data quality, we implemented multiple filtering criteria to eliminate genetic instruments of poor quality. Initially, we eliminated SNPs with inadequate strength (F-statistic < 10). Subsequently, after aligning the summary data for exposure and outcome, we chose SNPs as instrumental variables that were conditionally independent and showed no linkage disequilibrium (r^2^ < 0.1, according to the 1000 Genomes European reference panel). Additionally, we employed Steiger filtering to exclude genes where the trait's variance exceeded that of the exposure (refer to Table S2 for details).

MR estimates were computed using the Wald ratio approach for every SNP in the initial analysis. After performing the calculation of MR estimates for SNPs, the IVW, MR-Egger, and weighted median models were used for meta-analysis, all of which utilized multiple instruments. When proposed instruments included more than two variations, as a means of addressing potential pleiotropy in the relationships between the relevant exposure and the outcomes, MR-Egger regression was used. Cochran's Q test was utilized to evaluate heterogeneity among instrumental variables. To address the issue of multiple testing, we utilized Bonferroni adjustments to determine modified significance thresholds for the sensitivity analyses. In the IEU-B-4980, we established significance by considering p-values lower than 2.08E−5 (p = 0.05/2405). Subsequently, the IEU-B-69 dataset replicated targets that met this criterion. Significance was attributed to associations with p-values less than 0.01 (p = 0.05/5) in the replication analyses.

We performed a colocalization analysis to investigate the relationship between MR findings that were statistically significant in both cohorts and the risk of sepsis. The analysis was conducted utilizing the coloc R package, employing the default priors. Within the eQTL dataset, we established the prior likelihood for cis-eQTL (H1) and sepsis connections (H2) as 1E−04, whereas the prior probability of a solitary variation influencing both characteristics (H4) was designated as 1E−05. We deemed colocalization to be significant if the posterior probability (PPH4) was greater than 0.70. Genes displaying strong colocalization with sepsis were identified as potential molecular targets.

## Results

### Study design

**Figure 1:**
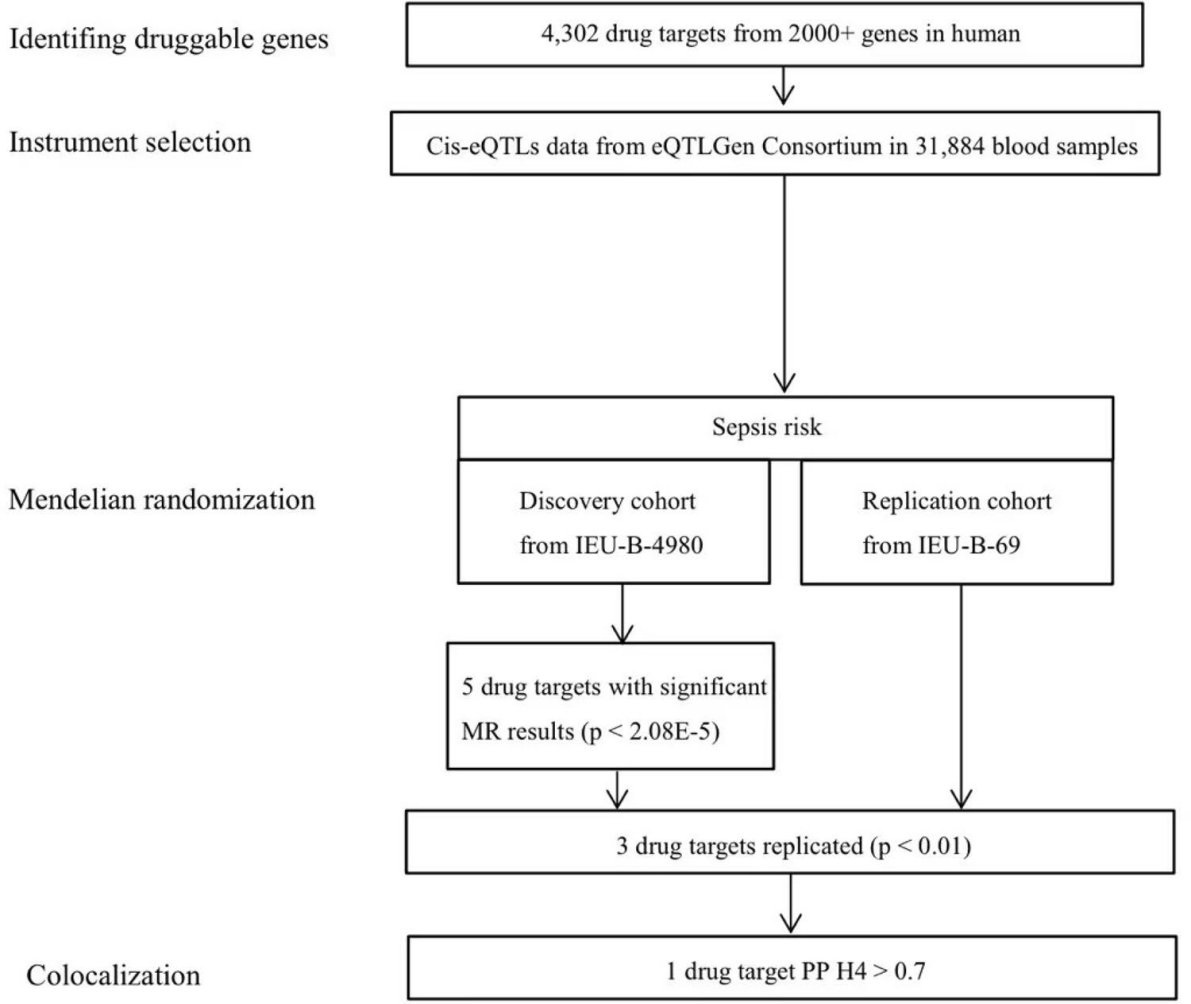
Overview of the study design in our Mendelian randomization study.

Our study endeavors to pinpoint therapeutic targets associated with sepsis. The methodology is succinctly outlined in Fig. 1. At first, we recognized 4302 unique human genes that encode proteins and can be targeted by drugs^9^. Afterward, we chose conditionally independent cis-eQTL variants that are closely related to gene expression. Next, we explored the significance of therapeutic target mRNA expression in relation to the risk of sepsis by utilizing a two-sample MR method. We conducted colocalization analysis for MR outcomes that reached the significance threshold, accounted for multiple tests, and were later validated in a secondary cohort. The objective of this analysis was to determine if the MR findings were affected by different causal mutants that were in a state of linkage disequilibrium with one another.

### Discovery analysis

By employing cis-eQTL information obtained from the eQTLGen Consortium^18^, we carefully discovered 2405 targetable genes through clumping. Subsequently, using European summary data for sepsis patients, we conducted a two-sample MR analysis. Using the IVW meta-analysis technique, each genetic instrument's effect estimates in our discovery cohort were combined, encompassing 10,154 sepsis patients and 452,764 controls derived from the IEU-B-4980. Surprisingly, we discovered that the genetically anticipated levels of expression for 5 genes were linked to the risk of sepsis. This finding remained statistically significant even after thorough adjustment for multiple testing (p < 2.08E−5, equivalent to p = 0.05/2405 after IVW and a Bonferroni correction of 0.05 was applied to 2405 drug targets, as shown in Tables S3, S4).

### Replication analysis

Utilizing data obtained from the IEU-B-69, we aimed to reproduce the impact calculations for the five most significant genes that were identified in the initial stage. Satisfyingly, three of the drug targets, specifically PSMA4, IFNAR2, and LY9, demonstrated replication that exceeded a strict Bonferroni threshold (p < 0.01 [IVW], which is equivalent to 0.05/5 genes, as shown in Table 1 and Tables S5, S6). Furthermore, the effect direction remained consistent among these genes, demonstrating a complete agreement of 100%. Significantly, another gene, PDGFB, attained nominal significance (p < 0.05 [IVW]) in this replication endeavor.

**Table 1:** Mendelian randomization results.

Genes	IEU-B-4980					IEU-B-69				
	SNPs	OR	IVW p-value	MR-Egger intercept	Egger intercept p-value	SNPs	OR	IVW p-value	MR-Egger intercept	Egger intercept p-value
PSMA4	7	1.31843	1.66E−08	0.012708	0.612573	7	1.293275	1.38E−05	0.036965	0.206849
PDGFB	23	0.858718	4.68E−08	0.000749	0.940136	23	0.936504	0.023142	0.008593	0.39847
IFNAR2	28	1.151974	1.26E−07	0.006544	0.406202	28	1.110615	0.00016	− 0.01998	0.019715
LY9	13	0.813229	1.00E−05	− 0.01768	0.166828	13	0.871143	0.004541	− 0.01089	0.398483
SERPINE2	36	0.938769	1.75E−05	− 0.01363	0.148345	36	0.998553	0.926654	− 0.0099	0.322391

### Colocalization analysis

**Figure 2:**
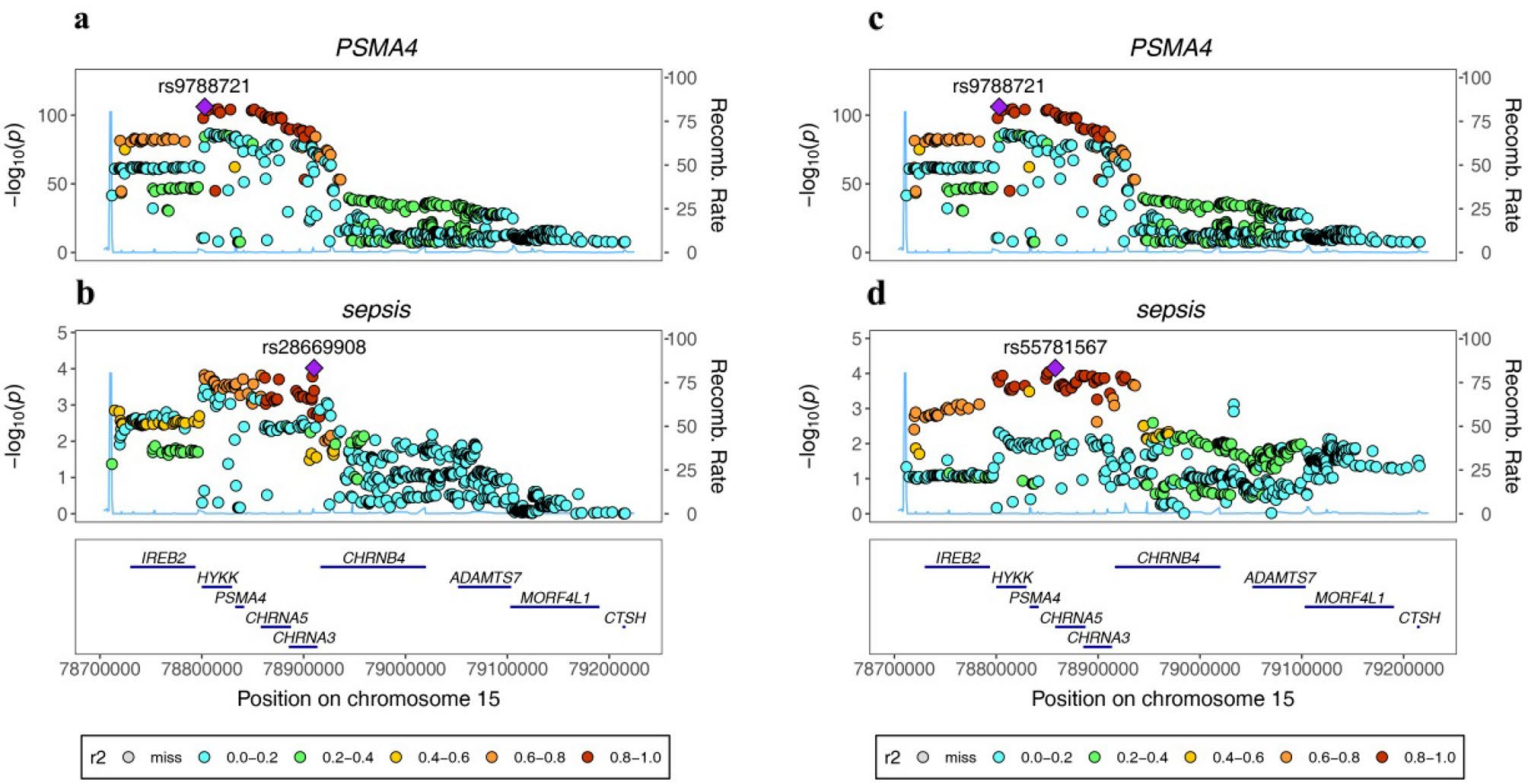
Regional Manhattan plot of associations of SNPs with PSMA4 locus. (**a**) In the IEU-B-4980 dataset rs9788721 was used to represent serum PSMA4 expression. (**b**) In the IEU-B-4980 dataset rs28669908 and its flanking 400 kb region to either side in sepsis. (**c**) In the IEU-B-69 dataset rs9788721 was used to proxy serum PSMA4 expression. (**d**) In the IEU-B-69 dataset rs55781567 and its flanking 400 kb region to either side in aortic aneurysm.

To further investigate the probability of SNPs linked to sepsis and eQTL sharing causal genetic variations, we performed a colocalization analysis. The results of this analysis suggested a likely common causal variant in the PSMA4 region between PSMA4 and sepsis (PP.H4 = 0.74, Fig. 2). As a result, we have discovered a gene that may be targeted by drugs, supported by strong evidence of a common genetic impact on both eQTL and sepsis risk, which was determined through a combination of MR and colocalization analyses (Table S7).

### PSMA4

The MR results for PSMA4 revealed a positive estimate effect, implying a correlation between heightened PSMA4 manifestation and heightened susceptibility to sepsis (OR 1.32, 95% CI 1.20–1.45). Consequently, the use of PSMA4 antagonists could represent an innovative approach to mitigating the risk of sepsis. Our validation of PSMA4 colocalization in the IEU-B-69 further underscores its potential as a therapeutic target for sepsis (PP.H4 = 0.85). Notably, Cochran's Q-test statistics did not indicate heterogeneity in the case of PSMA4 (P = 0.39). Additionally, we employed the MR Egger intercept to assess PSMA4 pleiotropy, which pertains to the phenomenon where a single genetic variant can impact multiple traits, thereby violating the fundamental assumption of being a valid IV. The results revealed a p-value exceeding 0.05 (p = 0.61), signifying the absence of significant directional pleiotropy in PSMA4.

## Discussion

Investigating potentially druggable genes that could serve as protective factors against sepsis, we conducted an extensive MR analysis by integrating GWAS datasets, drug genome information, and gene expression data (eQTL and pQTL). Through the utilization of different meta-analysis techniques like IVW, MR-Egger, and other supplementary examinations such as the MR-Egger intercept test, Cochran's Q test, pQTL investigations, and colocalization analyses, we have successfully discovered a robust drug target PSMA4 with substantial MR support.

PSMA4, which is accountable for encoding subunits of the proteasome, has a pivotal function in the regulation of inflammation, transduction of signaling pathways, and response to stress^20,21^. Knocking down PSMA4 has been shown to reduce proteasome activity and lead to the accumulation of ubiquitinated proteins in vitro^22,23^. The proteasome has a hand in controlling numerous cellular processes, including transcription, cell cycle progression, and apoptosis^24,25^. In our study, we found a significant association between PSMA4 and sepsis risk (IEU-B-4980: OR 1.32, p = 1.66E−08; IEU-B-69: OR 1.29, p = 1.38E−05). Furthermore, powerful evidence for colocalization of PSMA4 with sepsis was detected (PP.H4 = 0.74).

Proteasome inhibitors are known to modulate excessive inflammatory and immune responses and regulate cytokine expression triggered by various stimuli^26–28^. Their potent anti-inflammatory effects primarily result from the reduction of NF-κB activation. Bortezomib, for instance, inhibits the chymotrypsin-like activity of the 26S proteasome complex in the ubiquitin–proteasome system (UPS) and was the first FDA-approved proteasome inhibitor for multiple myeloma treatment^29–31^. Sepsis, characterized by uncontrolled inflammation, is a consequence of NF-κB activation via intracellular signaling pathways like Toll-like receptors^32^. Studies have demonstrated that pretreatment with low concentrations of bortezomib (25 nM or 50 nM) increases survival in septic mice, reduces cytokine levels, and mitigates the inflammatory response^33^.

While there's growing understanding of sepsis pathogenesis, current treatments predominantly rely on antibiotics for infection control^34^. Sepsis patients experience different immune states during pathogenesis, and anti-cytokine or anti-inflammatory therapies should be tailored accordingly^35,36^. Many anti-inflammatory treatments targeting pro-inflammatory cytokines have failed, potentially due to a lack of consideration for the patient's immune status. Using anti-inflammatory drugs during the immunocompromised phase could worsen the condition^33,36^. Thus, the development of new therapeutic agents and strategies is essential to reduce the high mortality associated with severe sepsis. To guarantee reliable outcomes from MR analysis, we implemented the Bonferroni correction for multiple tests to minimize the possibility of incorrect positive results. To minimize the potential for biased outcomes caused by pleiotropy, we utilized various MR techniques that are robust to pleiotropy, along with outlier detection methods.

The database populations utilized in this analysis all consisted of individuals with European ancestry. However, it's worth noting that only three out of the five drug targets identified in the IEU-B-4980 were effectively validated in the IEU-B-69. This discrepancy might be attributed to the fact that contemporary Europeans originate from three distinct subgroups of genetic differentiation.

In this research, we have pioneered the application of Mendelian randomization to identify promising treatment targets for sepsis. By employing genomic methodologies, we have circumvented the limitations associated with traditional new drug development, leading to substantial time and cost savings. Our study has pinpointed PSMA4 as a significant drug target for sepsis through MR analysis. Additionally, we have highlighted specific small molecule inhibitors currently in development for PSMA4, offering valuable insights for future drug development efforts focused on this target.

This study presents several limitations. Firstly, we did not assess the safety profile or potential alternative applications of the identified targets. Furthermore, the absence of experiments involving animals or clinical samples hinders the accessibility of real-world evidence to substantiate our MR analysis findings. In conclusion, our research sample solely comprised individuals of European descent, thereby limiting the applicability of our results to other ethnicities.

To summarize, this research offers evidence for the possibility of decreasing the likelihood of sepsis by targeting PSMA4. Nonetheless, it is crucial to conduct randomized experiments to evaluate the efficacy and security of sepsis therapy.

### Supplementary Information

Supplementary Tables.

## Data Availability

All of the original GWAS files are available for download.

## Abbreviations

IVW: Inverse variance weighted
GWAS: Genome-wide association studies
MR: Mendelian randomization
OR: Odds ratio
SNP: Single nucleotide polymorphism
cis-eQTL: Cis-expression quantitative trait loci
ICD: International Classification of Diseases
GBD: Global Burden of Disease
UPS: Ubiquitin–proteasome system
