# Supplementary material for: Using Mendelian randomization provides genetic insights into potential targets for sepsis treatment
Source: Sci Rep. 2024 Apr 11;14:8467. doi: 10.1038/s41598-024-58457-1 (PMC11009318; doi:10.1038/s41598-024-58457-1)
Supplement: Supplementary file 1 — Supplementary Tables. [file 41598_2024_58457_MOESM1_ESM.docx]

Table S1. Data sources and corresponding ICD codes for sepsis in our study

| **Source** | **Sample size, N** | **Case, N** | **Control, N** | **Ancestry** |
| --- | --- | --- | --- | --- |
| IEU-B-4980 | 486,484 | 11,643 | 474,841 | European |
| IEU-B-69 | 462,918 | 10,154 | 454,764 | European |

Table S2. A detailed summary of genetic variants in 5 significant genes

| **Gene** | **SNP** | **Effect_allele.exposure** | **Other_allele.exposure** | **Beta** | **se** | **Eaf** | **P-value** | R2 | **F-statistic** |
| --- | --- | --- | --- | --- | --- | --- | --- | --- | --- |
| PSMA4 | rs1052035 | C | T | 0.319835530603225 | 0.0210864812697441 | 0.0427856885364095 | 5.8E-52 | 6.95E-03 | 230.0622 |
| PSMA4 | rs12442456 | T | G | -0.126674156830225 | 0.0106089593084114 | 0.173326 | 7.29E-33 | 4.32E-03 | 142.5708 |
| PSMA4 | rs12914822 | A | G | -0.0835953464885232 | 0.00992123648376116 | 0.25712 | 3.58E-17 | 2.16E-03 | 70.99579 |
| PSMA4 | rs147760547 | G | A | 0.223223244348397 | 0.0388065860624451 | 0.018243 | 8.82E-09 | 1.01E-03 | 33.0878 |
| PSMA4 | rs74386627 | T | C | -0.154115724575075 | 0.0267617775534963 | 0.031545 | 8.46E-09 | 1.01E-03 | 33.16378 |
| PSMA4 | rs76474922 | C | A | -0.186233448910476 | 0.0162824211957365 | 0.071108 | 2.71E-30 | 3.96E-03 | 130.821 |
| PSMA4 | rs77802411 | C | T | 0.139358087147332 | 0.023939341237753 | 0.035289 | 5.84E-09 | 1.03E-03 | 33.88753 |
| PSMA4 | rs931794 | G | A | 0.183504260974658 | 0.00835264460776064 | 0.66408 | 5.6E-107 | 1.45E-02 | 482.6633 |
| PDGFB | rs112931952 | T | C | -0.19879 | 0.024991 | 0.027836 | 1.79E-15 | 1.92E-03 | 63.27725 |
| PDGFB | rs117067023 | T | C | -0.1918 | 0.024179 | 0.032626 | 2.15E-15 | 1.91E-03 | 62.92456 |
| PDGFB | rs117102928 | A | G | -0.27291 | 0.029322 | 0.020914 | 1.31E-20 | 2.63E-03 | 86.62397 |
| PDGFB | rs117138710 | C | T | 0.140438 | 0.027638 | 0.023947 | 3.75E-07 | 7.85E-04 | 25.82063 |
| PDGFB | rs12483986 | T | C | -0.14632 | 0.013118 | 0.103361 | 6.86E-29 | 3.77E-03 | 124.4073 |
| PDGFB | rs137590 | C | T | 0.08858 | 0.008637 | 0.572358 | 1.12E-24 | 3.19E-03 | 105.1753 |
| PDGFB | rs139372 | T | C | -0.07701 | 0.009316 | 0.241837 | 1.38E-16 | 2.07E-03 | 68.33668 |
| PDGFB | rs141193191 | T | C | -0.16121 | 0.035864 | 0.017816 | 6.95E-06 | 6.14E-04 | 20.20592 |
| PDGFB | rs150210284 | T | C | -0.173 | 0.02495 | 0.031611 | 4.1E-12 | 1.46E-03 | 48.07758 |
| PDGFB | rs34963492 | C | A | -0.13717 | 0.024985 | 0.030494 | 4.01E-08 | 9.16E-04 | 30.1401 |
| PDGFB | rs4820372 | G | A | -0.15114 | 0.011217 | 0.146022 | 2.22E-41 | 5.49E-03 | 181.5514 |
| PDGFB | rs56133813 | C | T | 0.246043 | 0.04596 | 0.019889 | 8.64E-08 | 8.71E-04 | 28.65889 |
| PDGFB | rs5750769 | T | C | 0.098563 | 0.011495 | 0.140011 | 9.94E-18 | 2.23E-03 | 73.52548 |
| PDGFB | rs5750780 | A | G | -0.22696 | 0.024316 | 0.032332 | 1.02E-20 | 2.64E-03 | 87.11982 |
| PDGFB | rs5757502 | A | T | 0.080144 | 0.008803 | 0.29091 | 8.72E-20 | 2.52E-03 | 82.87917 |
| PDGFB | rs5757572 | C | G | -0.35531 | 0.008677 | 0.356165 | 3.3E-303 | 4.85E-02 | 1676.64 |
| PDGFB | rs73161596 | A | G | 0.203821 | 0.033316 | 0.021384 | 9.49E-10 | 1.14E-03 | 37.4287 |
| PDGFB | rs73163486 | T | C | 0.177302 | 0.033279 | 0.020243 | 9.94E-08 | 8.63E-04 | 28.38545 |
| PDGFB | rs73424776 | C | G | -0.36713 | 0.028981 | 0.028079 | 8.87E-37 | 4.86E-03 | 160.4808 |
| PDGFB | rs76002309 | T | C | 0.082063 | 0.015639 | 0.081407 | 1.54E-07 | 8.37E-04 | 27.53311 |
| PDGFB | rs77004047 | T | C | 0.112092 | 0.02135 | 0.042829 | 1.52E-07 | 8.38E-04 | 27.56565 |
| PDGFB | rs77379297 | A | G | 0.089525 | 0.00865 | 0.69755 | 4.2E-25 | 3.25E-03 | 107.1163 |
| PDGFB | rs77674834 | A | G | 0.191782 | 0.027545 | 0.024953 | 3.35E-12 | 1.47E-03 | 48.47501 |
| PDGFB | rs9622983 | T | C | 0.152106 | 0.011915 | 0.865411 | 2.53E-37 | 4.93E-03 | 162.981 |
| IFNAR2 | rs117407002 | G | A | 0.152661 | 0.024478 | 0.032657 | 4.47E-10 | 1.18E-03 | 38.89518 |
| IFNAR2 | rs118080354 | A | C | -0.2305 | 0.042766 | 0.025166 | 7.05E-08 | 8.83E-04 | 29.04994 |
| IFNAR2 | rs1475836 | A | G | 0.194466 | 0.024077 | 0.966241 | 6.66E-16 | 1.98E-03 | 65.23308 |
| IFNAR2 | rs16990416 | C | T | 0.217856 | 0.020513 | 0.043522 | 2.4E-26 | 3.42E-03 | 112.795 |
| IFNAR2 | rs17860208 | G | A | 0.283662 | 0.022304 | 0.03943 | 4.7E-37 | 4.90E-03 | 161.7526 |
| IFNAR2 | rs17860257 | T | C | 0.208661 | 0.032795 | 0.019411 | 1.99E-10 | 1.23E-03 | 40.48141 |
| IFNAR2 | rs17860260 | T | C | 0.120122 | 0.027612 | 0.026813 | 1.36E-05 | 5.76E-04 | 18.92598 |
| IFNAR2 | rs17860261 | G | C | -0.41521 | 0.024189 | 0.03052 | 4.85E-66 | 8.89E-03 | 294.6372 |
| IFNAR2 | rs17860265 | A | G | 0.296329 | 0.027091 | 0.029101 | 7.59E-28 | 3.63E-03 | 119.642 |
| IFNAR2 | rs189110596 | G | A | -0.21555 | 0.027494 | 0.0266 | 4.5E-15 | 1.87E-03 | 61.46717 |
| IFNAR2 | rs2186277 | T | C | 0.347281 | 0.008182 | 0.576755 | 3.3E-303 | 5.20E-02 | 1801.553 |
| IFNAR2 | rs2229207 | C | T | -0.28858 | 0.016616 | 0.083127 | 1.46E-67 | 9.09E-03 | 301.6231 |
| IFNAR2 | rs2834136 | A | G | 0.17748 | 0.027368 | 0.024637 | 8.89E-11 | 1.28E-03 | 42.05393 |
| IFNAR2 | rs2834167 | G | A | -0.07535 | 0.009487 | 0.736053 | 1.98E-15 | 1.92E-03 | 63.08172 |
| IFNAR2 | rs2834185 | T | C | 0.078739 | 0.008784 | 0.339882 | 3.14E-19 | 2.44E-03 | 80.34792 |
| IFNAR2 | rs2843983 | A | C | 0.083916 | 0.009155 | 0.332415 | 4.92E-20 | 2.55E-03 | 84.01372 |
| IFNAR2 | rs34579880 | A | G | 0.216312 | 0.045658 | 0.016349 | 2.16E-06 | 6.83E-04 | 22.4458 |
| IFNAR2 | rs56027221 | G | A | -0.14048 | 0.024511 | 0.03258 | 9.95E-09 | 9.99E-04 | 32.84895 |
| IFNAR2 | rs62228039 | G | A | -0.06175 | 0.010221 | 0.770356 | 1.52E-09 | 1.11E-03 | 36.50456 |
| IFNAR2 | rs73205061 | A | T | -0.22003 | 0.027112 | 0.026408 | 4.83E-16 | 2.00E-03 | 65.86296 |
| IFNAR2 | rs764278 | A | G | -0.09976 | 0.010444 | 0.197762 | 1.27E-21 | 2.77E-03 | 91.23497 |
| IFNAR2 | rs76681806 | A | G | -0.10569 | 0.024785 | 0.030547 | 2E-05 | 5.53E-04 | 18.18511 |
| IFNAR2 | rs77224013 | A | G | 0.129987 | 0.018544 | 0.053684 | 2.39E-12 | 1.49E-03 | 49.1373 |
| IFNAR2 | rs78786709 | A | G | -0.18048 | 0.029791 | 0.022552 | 1.37E-09 | 1.12E-03 | 36.70179 |
| IFNAR2 | rs78940750 | A | G | -0.24696 | 0.038944 | 0.015516 | 2.28E-10 | 1.22E-03 | 40.21209 |
| IFNAR2 | rs8132172 | A | G | 0.125266 | 0.009446 | 0.737883 | 3.85E-40 | 5.32E-03 | 175.8806 |
| IFNAR2 | rs8178434 | G | T | -0.17014 | 0.039022 | 0.014988 | 1.3E-05 | 5.78E-04 | 19.0096 |
| IFNAR2 | rs8178561 | A | G | 0.100185 | 0.019514 | 0.050418 | 2.84E-07 | 8.01E-04 | 26.35693 |
| IFNAR2 | rs9974928 | G | A | 0.06575 | 0.011965 | 0.172931 | 3.91E-08 | 9.18E-04 | 30.19722 |
| IFNAR2 | rs9984273 | C | T | 0.046421 | 0.009023 | 0.31191 | 2.68E-07 | 8.05E-04 | 26.46794 |
| LY9 | rs114458235 | C | T | -0.30649 | 0.037775 | 0.014606 | 4.91E-16 | 2.00E-03 | 65.82888 |
| LY9 | rs114765928 | A | G | -0.26077 | 0.03354 | 0.021355 | 7.53E-15 | 1.84E-03 | 60.45063 |
| LY9 | rs12072397 | A | T | 0.10867 | 0.009124 | 0.314219 | 1.05E-32 | 4.30E-03 | 141.841 |
| LY9 | rs12131917 | A | G | -0.1119 | 0.026168 | 0.029997 | 1.9E-05 | 5.56E-04 | 18.28589 |
| LY9 | rs141204272 | G | A | -0.27349 | 0.040127 | 0.018944 | 9.39E-12 | 1.41E-03 | 46.45104 |
| LY9 | rs16832364 | C | A | -0.23076 | 0.026742 | 0.024967 | 6.17E-18 | 2.26E-03 | 74.46309 |
| LY9 | rs182137476 | A | G | -0.30487 | 0.060683 | 0.028138 | 5.06E-07 | 7.67E-04 | 25.24058 |
| LY9 | rs2295616 | A | G | 0.06509 | 0.008725 | 0.299706 | 8.64E-14 | 1.69E-03 | 55.65458 |
| LY9 | rs4656932 | A | G | -0.2467 | 0.009557 | 0.784246 | 6.4E-147 | 1.99E-02 | 666.2903 |
| LY9 | rs500188 | T | C | -0.06777 | 0.008328 | 0.633187 | 4.03E-16 | 2.01E-03 | 66.22216 |
| LY9 | rs6691868 | C | T | 0.086644 | 0.008915 | 0.656855 | 2.5E-22 | 2.87E-03 | 94.46479 |
| LY9 | rs67841898 | A | G | 0.107776 | 0.021448 | 0.037999 | 5.04E-07 | 7.68E-04 | 25.24962 |
| LY9 | rs72710801 | A | C | -0.20244 | 0.045761 | 0.013142 | 9.69E-06 | 5.95E-04 | 19.57089 |
| LY9 | rs80242873 | T | C | 0.125339 | 0.026273 | 0.027415 | 1.84E-06 | 6.92E-04 | 22.75862 |
| LY9 | rs806570 | T | C | 0.079323 | 0.010231 | 0.293932 | 8.99E-15 | 1.83E-03 | 60.10901 |
| SERPINE2 | rs10187546 | T | G | 0.181552 | 0.008528 | 0.675666 | 1.4E-100 | 1.36E-02 | 453.2556 |
| SERPINE2 | rs10191160 | G | A | -0.1356 | 0.010983 | 0.160521 | 5.09E-35 | 4.62E-03 | 152.4311 |
| SERPINE2 | rs10192864 | A | G | 0.095813 | 0.008112 | 0.43257 | 3.39E-32 | 4.23E-03 | 139.521 |
| SERPINE2 | rs10206895 | A | T | 0.398044 | 0.008783 | 0.274539 | 3.3E-303 | 5.88E-02 | 2053.766 |
| SERPINE2 | rs114151942 | A | G | 0.541722 | 0.027231 | 0.027628 | 4.62E-88 | 1.19E-02 | 395.7593 |
| SERPINE2 | rs114211301 | G | T | -0.23583 | 0.021605 | 0.037716 | 9.71E-28 | 3.61E-03 | 119.1503 |
| SERPINE2 | rs114331540 | T | C | -0.50205 | 0.101903 | 0.014231 | 8.35E-07 | 7.38E-04 | 24.27336 |
| SERPINE2 | rs114750941 | A | G | -0.25656 | 0.031168 | 0.022034 | 1.84E-16 | 2.06E-03 | 67.75924 |
| SERPINE2 | rs115831753 | T | G | 0.490861 | 0.066661 | 0.016044 | 1.79E-13 | 1.65E-03 | 54.22113 |
| SERPINE2 | rs115892774 | A | G | -0.44682 | 0.079392 | 0.015086 | 1.82E-08 | 9.63E-04 | 31.67438 |
| SERPINE2 | rs115955266 | G | A | 0.256563 | 0.032274 | 0.017053 | 1.87E-15 | 1.92E-03 | 63.19455 |
| SERPINE2 | rs116008011 | A | G | -0.61195 | 0.031896 | 0.023495 | 4.86E-82 | 1.11E-02 | 368.0911 |
| SERPINE2 | rs116598593 | C | T | -0.57731 | 0.054822 | 0.015667 | 6.23E-26 | 3.36E-03 | 110.8935 |
| SERPINE2 | rs11695603 | A | G | 0.146058 | 0.030319 | 0.022123 | 1.46E-06 | 7.06E-04 | 23.20638 |
| SERPINE2 | rs11884404 | T | C | 0.35076 | 0.00862 | 0.706839 | 3.3E-303 | 4.80E-02 | 1655.912 |
| SERPINE2 | rs12478391 | A | C | -0.51579 | 0.034047 | 0.018058 | 7.65E-52 | 6.93E-03 | 229.5013 |
| SERPINE2 | rs13001654 | T | C | -0.13594 | 0.012572 | 0.117081 | 2.99E-27 | 3.55E-03 | 116.9145 |
| SERPINE2 | rs140405039 | T | C | -0.59896 | 0.036922 | 0.017142 | 3.5E-59 | 7.94E-03 | 263.1598 |
| SERPINE2 | rs141186096 | A | C | 0.296098 | 0.039177 | 0.017033 | 4.09E-14 | 1.74E-03 | 57.12336 |
| SERPINE2 | rs145554607 | T | C | 0.098853 | 0.022918 | 0.033183 | 1.61E-05 | 5.66E-04 | 18.60542 |
| SERPINE2 | rs145716019 | T | C | -0.50719 | 0.037516 | 0.015975 | 1.2E-41 | 5.53E-03 | 182.7769 |
| SERPINE2 | rs146209210 | T | G | -0.23884 | 0.031376 | 0.02067 | 2.69E-14 | 1.76E-03 | 57.94559 |
| SERPINE2 | rs147808525 | T | C | 0.875239 | 0.112899 | 0.01113 | 9.02E-15 | 1.83E-03 | 60.09971 |
| SERPINE2 | rs17196910 | T | C | 0.549492 | 0.026399 | 0.026722 | 3.15E-96 | 1.30E-02 | 433.2726 |
| SERPINE2 | rs2123714 | C | T | 0.069033 | 0.009465 | 0.235597 | 3.03E-13 | 1.62E-03 | 53.19077 |
| SERPINE2 | rs2438171 | T | G | 0.400149 | 0.008523 | 0.452413 | 3.3E-303 | 6.29E-02 | 2204.425 |
| SERPINE2 | rs62185746 | T | G | -0.12592 | 0.020202 | 0.047151 | 4.57E-10 | 1.18E-03 | 38.85154 |
| SERPINE2 | rs62188374 | A | C | -0.3344 | 0.039278 | 0.015996 | 1.68E-17 | 2.20E-03 | 72.48138 |
| SERPINE2 | rs650137 | G | A | 0.164808 | 0.017277 | 0.058184 | 1.44E-21 | 2.76E-03 | 90.99252 |
| SERPINE2 | rs6712954 | A | G | 0.259802 | 0.018516 | 0.049844 | 1E-44 | 5.95E-03 | 196.8802 |
| SERPINE2 | rs72958579 | T | C | 0.18468 | 0.020226 | 0.043824 | 6.81E-20 | 2.53E-03 | 83.37151 |
| SERPINE2 | rs74807729 | A | G | 0.4641 | 0.032013 | 0.018667 | 1.26E-47 | 6.35E-03 | 210.1746 |
| SERPINE2 | rs7580849 | T | C | 0.22456 | 0.024519 | 0.040581 | 5.27E-20 | 2.55E-03 | 83.87812 |
| SERPINE2 | rs77310158 | A | G | 0.146 | 0.032833 | 0.032984 | 8.72E-06 | 6.01E-04 | 19.77403 |
| SERPINE2 | rs77883969 | A | C | 0.261571 | 0.033357 | 0.016245 | 4.46E-15 | 1.87E-03 | 61.48912 |
| SERPINE2 | rs78106908 | A | C | -0.29023 | 0.062666 | 0.021778 | 3.63E-06 | 6.52E-04 | 21.44987 |
| SERPINE2 | rs79580836 | C | A | -0.36672 | 0.009812 | 0.203824 | 1E-303 | 4.08E-02 | 1396.831 |

Table S3. Mendelian randomization analysis results in IEU-B-4980

| **Gene** | **Method** | **SNP(n)** | **b** | **se** | **P-value** | **OR** | **OR_LCI95** | **OR_UCI95** |
| --- | --- | --- | --- | --- | --- | --- | --- | --- |
| PSMA4 | Inverse variance weighted | 7 | 0.276442 | 0.048983 | 1.66E-08 | 1.31843 | 1.197739 | 1.451283 |
| PDGFB | Inverse variance weighted | 23 | -0.15231 | 0.027881 | 4.68E-08 | 0.858718 | 0.813052 | 0.90695 |
| IFNAR2 | Inverse variance weighted | 28 | 0.141477 | 0.026768 | 1.26E-07 | 1.151974 | 1.093092 | 1.214027 |
| LY9 | Inverse variance weighted | 13 | -0.20674 | 0.046813 | 1.00E-05 | 0.813229 | 0.741934 | 0.891376 |
| SERPINE2 | Inverse variance weighted | 36 | -0.06319 | 0.014713 | 1.75E-05 | 0.938769 | 0.912084 | 0.966234 |

Table S4. The results of pleiotropy analysis in IEU-B-4980

| **Exposure** | **MR-Egger intercept** | **MR-Egger SE** | **MR-Egger P-value** |
| --- | --- | --- | --- |
| PSMA4 | 0.012708 | 0.023548 | 0.612573 |
| PDGFB | 0.000749 | 0.009854 | 0.940136 |
| IFNAR2 | 0.006544 | 0.00775 | 0.406202 |
| LY9 | -0.01768 | 0.011942 | 0.166828 |
| SERPINE2 | -0.01363 | 0.009215 | 0.148345 |

Table S5. Mendelian randomization analysis results in IEU-B-69

| **Gene** | **Method** | **SNP(n)** | **b** | **se** | **P-value** | **OR** | **OR_LCI95** | **OR_UCI95** |
| --- | --- | --- | --- | --- | --- | --- | --- | --- |
| PSMA4 | Inverse variance weighted | 7 | 0.257178 | 0.059172 | 1.38E-05 | 1.293275 | 1.151656 | 1.45231 |
| PDGFB | Inverse variance weighted | 23 | -0.0656 | 0.028886 | 0.023142 | 0.936504 | 0.884956 | 0.991055 |
| IFNAR2 | Inverse variance weighted | 28 | 0.104914 | 0.027788 | 0.00016 | 1.110615 | 1.051743 | 1.172782 |
| LY9 | Inverse variance weighted | 13 | -0.13795 | 0.04861 | 0.004541 | 0.871143 | 0.791976 | 0.958223 |
| SERPINE2 | Inverse variance weighted | 36 | -0.00145 | 0.015733 | 0.926654 | 0.998553 | 0.96823 | 1.029825 |

Table S6. The results of pleiotropy analysis in IEU-B-69

| **Exposure** | **MR-Egger intercept** | **MR-Egger SE** | **MR-Egger P-value** |
| --- | --- | --- | --- |
| PSMA4 | 0.036965 | 0.0255 | 0.206849 |
| PDGFB | 0.008593 | 0.00997 | 0.39847 |
| IFNAR2 | -0.01998 | 0.008041 | 0.019715 |
| LY9 | -0.01089 | 0.012394 | 0.398483 |
| SERPINE2 | -0.0099 | 0.009854 | 0.322391 |

Table S7. The results of colocalization analysis

| Gene | Outcomes | PP.H0.abf | PP.H1.abf | PP.H2.abf | PP.H3.abf | PP.H4.abf |
| --- | --- | --- | --- | --- | --- | --- |
| PSMA4 | Sepsis | 1.50E-100 | 0.188660922 | 5.63E-101 | 0.070075341 | 0.741263736 |
| PDGFB | Sepsis | 0 | 0.305909 | 0 | 0.035135 | 0.658956 |
| LY9 | Sepsis | 5.60E-140 | 0.592185 | 2.39E-141 | 0.024949 | 0.382866 |
